# An Analysis of the Genetic Diversity, Genetic Structure, and Selection Signal of Beagle Dogs Using SNP Chips

**DOI:** 10.3390/genes16040358

**Published:** 2025-03-21

**Authors:** Haolong Wang, Yanbo Yin, Can Zhang, Fangzheng Li, Haiping Zhao, Zhen Liu, Weili Sun, Lisheng Zhou

**Affiliations:** 1 College of Animal Science and Technology, Qingdao Agricultural University, Qingdao 266109, China; wanghaolong725@163.com (H.W.); sunweili@caas.cn (W.S.); 2 College of Veterinary Medicine, Qingdao Agricultural University, Qingdao 266109, China

**Keywords:** beagle dogs, SNP chips, genetic diversity, genetic structure, selection signals

## Abstract

Background: Beagle dogs are widely used in biomedical research, but their genetic diversity and population structure require further investigation. This study aimed to assess genetic diversity, population structure, and selection signals in a foundational Beagle breeding population using genome-wide SNP genotyping. Methods: A total of 459 Beagle dogs (108 males, 351 females) were genotyped using the Canine 50K SNP chip. After quality control, 456 individuals and 31,198 SNPs were retained. Genetic diversity indices, principal component analysis (PCA), identity-by-state (IBS) distance, a genomic relationship matrix (G-matrix), runs of homozygosity (ROH), and Tajima’s D selection scans were analyzed. Results: The average minor allele frequency was 0.224, observed heterozygosity was 0.303, and expected heterozygosity was 0.305. A total of 2990 ROH segments were detected, with a mean inbreeding coefficient of 0.031. Phylogenetic analysis classified 106 stud dogs into 13 lineages. Selection signal analysis identified *TTN* (muscle function) and *DLA-DRA*, *DLA-DOA*, *DLA-DMA* (immune regulation) under selection. Conclusions: The Beagle population exhibits high genetic diversity and low inbreeding. To maintain genetic stability and ensure the long-term conservation of genetic resources, structured breeding strategies should be implemented based on lineage classifications.

## 1. Introduction

The Beagle, a medium-sized hunting dog originating in the United Kingdom, is celebrated for its remarkable olfactory senses and hunting capabilities. Known for its lively and cheerful temperament, friendliness towards humans, and distinctive tricolor coat of tan, black, and white with short, dense fur, the Beagle has become a popular breed worldwide [1]. Initially registered by the American Kennel Club (AKC) in 1885 [2], the breed was later acknowledged by the Fédération Cynologique Internationale (FCI) and the United Kennel Club (UKC) [3]. Beagles have consistently been listed among the top ten most popular dog breeds according to AKC rankings from 2013–2024 [4]. Their gentle temperament, minimal trait variation, and stable biological characteristics have also established them as an internationally preferred experimental animal [5], widely used in studies of physiology [6], pathology [7], and pharmacology [8].

In 1983, Beagles were officially introduced into China. Over the past four decades of breeding, closed populations adapted to domestic rearing conditions have been largely established [9]. However, the company Qingdao Bolong Experimental Animal Co., Ltd., which was established in the last decade, has developed a large-scale breeding program operating as an open breeding population. Challenges such as environmental influence, selection and breeding practices, and incomplete or missing pedigree records have resulted in limited knowledge of the genetic background and diversity within certain Beagle populations [10]. This deficiency hinders efforts towards population optimization and breed improvement.

Analyzing the genetic diversity and population structure of the foundational breeding population of experimental Beagle dogs at the molecular level is crucial for the effective utilization and conservation of their genetic resources. In addition to basic diversity analysis, identifying selection signals is equally important [11], as it can reveal key genes and pathways associated with physiological traits and adaptability in Beagle populations. Tajima’s D [12] has gained widespread attention for its application in dogs and other species [13,14]. Tajima’s D can detect both positive and balancing selection by examining the genetic variation in a population. A negative Tajima’s D value often indicates positive selection, where beneficial mutations increase in frequency and reduce genetic diversity. In contrast, a positive Tajima’s D value can indicate balancing selection, where multiple alleles are maintained in the population, often due to heterozygote advantage or frequency-dependent selection. Freedman employed Tajima’s D and Fst methods to detect characteristic genes shaped by selection in different dog populations and to examine genetic differences between dogs and wolves. Their study revealed that genes associated with neural function, morphology, and immune response were subject to positive selection. Notably, some of the genomic regions under study were shared across multiple dog breeds, while others were breed-specific. These findings highlight both the shared evolutionary history of dogs during domestication and the differentiation introduced by selective breeding practices [11].

Li conducted a study on the genetic diversity and phenotypic adaptability of several native dog breeds in China, using selection signal analysis. The study identified genes associated with pigmentation, high-altitude survival, skeletal structure, and ear morphology [15]. In canine genomics, Tajima’s D is frequently employed to pinpoint genomic regions associated with disease susceptibility, behavioral traits, and environmental adaptability [16]. For instance, Tajima’s D analysis in certain dog breeds has revealed key genes related to immune regulation, physical performance, and olfactory abilities—traits that are vital for canine physiology and dogs’ interactions with humans.

Genetic markers are instrumental in assessing genetic diversity within breeds and determining kinship among individuals [17]. Single nucleotide polymorphisms (SNPs), as third-generation molecular markers, offer advantages such as broad coverage, high specificity, and genetic stability. With the advancement of DNA microarray technology, the cost of large-scale genotyping has significantly decreased [18,19]. SNP chips have become a routine tool in genomic studies and are widely applied in livestock industries [20]. This study employs high-density genome-wide SNP genotyping chips to assess the genetic diversity, population structure, and selection signals of the foundational breeding population of experimental Beagles. The findings aim to provide valuable data for selective breeding, breed improvement, and the efficient utilization of genetic resources, while also identifying key genes and pathways that are critical to the Beagle’s physiological functions and environmental adaptability.

## 2. Materials and Methods

### 2.1. Animals

For this study, 459 Beagle dogs (108 males and 351 females), all aged 2–3 years, were selected from the basic breeding population of Qingdao Bolong Experimental Animal Co., Ltd., a major supplier of experimental animals in China. Blood samples were drawn into anticoagulant tubes and immediately stored at low temperatures to maintain sample quality. All procedures involving operations and animal care were authorized by the Experimental Animal Ethics Committee of Qingdao Agricultural University (approval number: DKY20230701).

### 2.2. Genotyping and Quality Control

TIANamp DNA Kits (Tiangen Biotech, Beijing, China) were used to extract genomic DNA from the blood samples. The concentration and purity were assessed with a NanoDrop^TM^ 2000 (Thermo Fisher, Waltham, MA, USA), and samples with an A260/280 ratio of 1.8–2.0 and a concentration above 50 ng/μL were deemed suitable for genotyping. All samples were genotyped using the Canine 50K SNP chip (Beijing Zhongke Kunpeng Biotechnology Co., Ltd., Beijing, China). Quality control was performed using PLINK v1.90 [21]. The following criteria were applied: SNPs with a minor allele frequency (MAF) below 0.01 were excluded, individuals with an SNP call rate exceeding 0.90 were retained, and those with a genotype call rate below 0.90 were removed [22].

### 2.3. Genetic Diversity Analysis

To comprehensively analyze the genetic diversity of the foundational breeding population, genetic diversity parameters were calculated using PLINK v1.90 [23], including the polymorphic marker ratio (P_N_), expected heterozygosity (H_E_), observed heterozygosity (H_O_), polymorphic information content (PIC), number of effective alleles (Ae), and minor allele frequency (MAF).

### 2.4. Genetic Relationships and Population Structure Analysis

Principal component analysis (PCA) was performed on quality control-filtered data using PLINK (v1.90). R software (v4.3.3) was employed for visualizing the population structure and stratification within the Beagle population. The identity-by-state (IBS) distance matrix and individual genetic distances were calculated using PLINK (v1.90). A G matrix was constructed using GCTA software (v1.94) [24] to evaluate genetic correlations [25]. Heatmaps for IBS and G matrices were visualized with R software. A phylogenetic tree was generated using the neighbor-joining method in TASSEL 5 [26], based on the IBS distance matrix. The phylogenetic tree was visualized with iTOL software (v6) [27]. For lineage analysis, we focused on male dogs (stud dogs) because they play a dominant role in genetic management due to their ability to sire multiple litters. By analyzing male lineages, we can better understand the genetic contributions of individual sires and develop targeted breeding strategies to maintain population health and diversity.

### 2.5. Inbreeding Coefficient Analysis

PLINK (v1.90) was used for genome-wide scanning of long runs of homozygosity (ROH), analyzing their distribution and count [28]. The inbreeding coefficient (*F_ROH_*) was calculated as the proportion of the total *ROH* segment length to the autosomal genome length using the following formula:

$$F_{ROH}=\frac{\sum_{N}Length\left(ROH_{N}\right)}{L}$$

where *N* represents the number of ROH segments, while *L* denotes the total length of the autosomal genome (Canis lupus familiaris Reference Genome Assembly 3.1). *R* was used to visualize *ROH* distributions and generate violin plots from *F_ROH_* values.

### 2.6. Selection Signal Analysis and Functional Annotation

To identify selection signals, sliding window analysis was conducted on the quality control-filtered data using Vcftools v0.1.6, with the window size set to 10 kb [29]. Tajima’s D values were computed for each window and ranked in ascending order. The top 1% of regions (threshold: −0.679435) were identified as undergoing significant positive selection. Gene annotation was performed using the CanFam3.1 canine reference genome and its corresponding annotation file. The GALLO package in R was utilized for this purpose [30]. From the Tajima’s D results, the top 10 genomic regions under positive selection were selected, along with genes situated within 500 kb on either side of each region.

Functional enrichment analysis of these genes was performed using the DAVID database (https://davidbioinformatics.nih.gov/; accessed on 15 January 2025) [31]. Gene Ontology (GO) terms and Kyoto Encyclopedia of Genes and Genomes (KEGG) pathways were analyzed to investigate the biological functions and metabolic pathways related to these genes. The most significant GO terms and KEGG pathways (*p* < 0.01) were selected for further analysis. A summary chart of the classifications was generated and visualized using the online platform (http://www.bioinformatics.com.cn/) [32].

## 3. Results

### 3.1. Genetic Diversity Analysis

**Figure 1:**
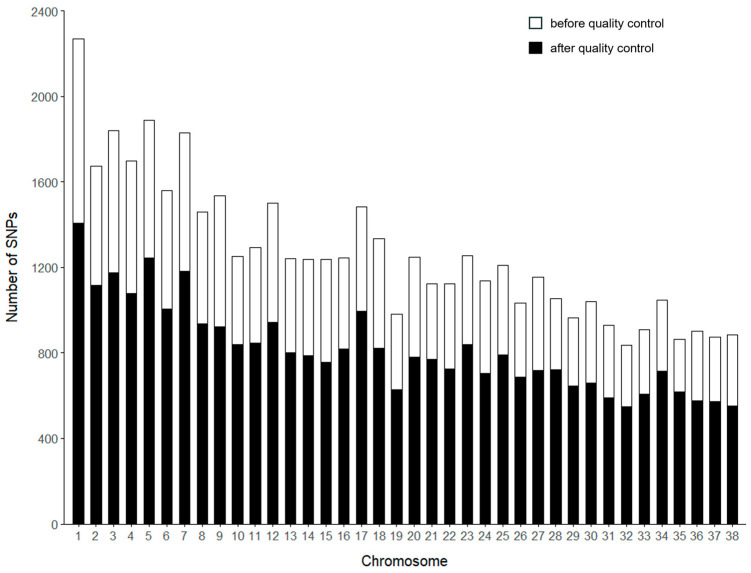
The number of SNPs per chromosome before and after quality control. The *x*-axis represents the chromosome number, while the *y*-axis indicates the number of SNPs. The white bars show the number of SNPs before quality control, while the black bars represent the SNPs retained after quality control.

A total of 48,304 SNPs were detected through genotyping. After quality control, 456 individuals (106 males and 350 females) and 31,198 SNPs remained for further analysis (Table 1). Before and after quality control, chromosome 1 had the highest number of SNPs, with 1409, while chromosome 32 had the lowest, with 550 (Figure 1). The genetic diversity of Beagle dogs was analyzed using PLINK (v1.90) software (Table 2). The analysis included an effective number of alleles (Ae) of 1.513, a minor allele frequency (MAF) of 0.224, observed heterozygosity (H_O_) of 0.303, expected heterozygosity (H_E_) of 0.305, and an average polymorphism information content (PIC) of 0.305. The majority of SNPs (66.1% with MAF ≥ 0.2) are common variants. Despite the inherent ascertainment bias in SNP array design favoring common variants, these markers still provide valuable insights into genetic diversity and selection signals, as they are less affected by missing data and genotyping errors compared to rare variants (Figure S1). These findings suggest that this Beagle dog population exhibits significant genetic diversity.

### 3.2. Population Structure Analysis

**Figure 2:**
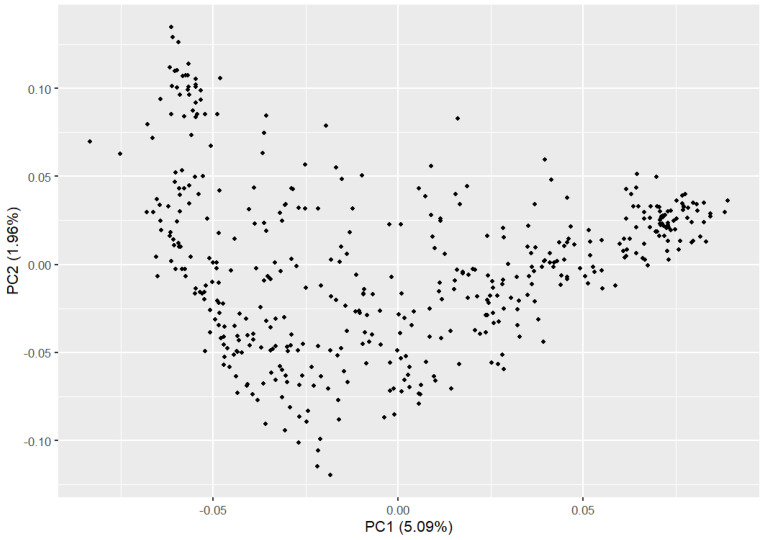
The population structure of the Beagle dogs.

**Figure 3:**
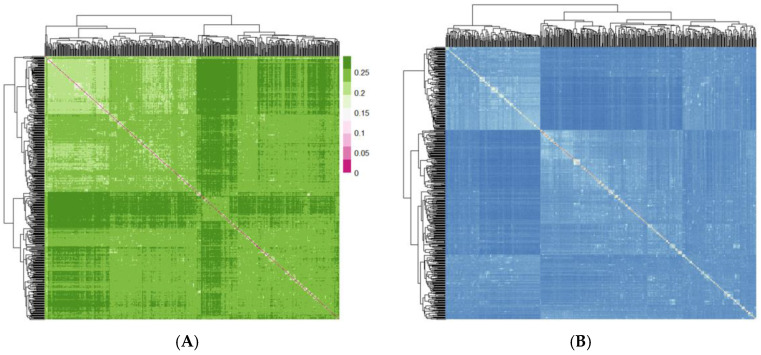
(**A**) Genetic relationship visualization based on the IBS distance matrix; (**B**) genetic relationship visualization based on the G matrix.

PCA results indicated a relatively dispersed population structure (Figure 2). Principal components 1 and 2 explained 5.09% and 1.96% of the variance, respectively, with no significant population stratification observed. We utilized PLINK (v1.90) to compute the IBS genetic distance between individuals to analyze the genetic relatedness within the Beagle dog population being studied (Figure 3A). The IBS genetic distance within the Beagle dog population ranges from 0.006 to 0.289, with an average of 0.245. Most individuals have relatively high IBS distances (shown in dark green), indicating a moderate genetic relationship, while some exhibit closer distances (shown in white). To further validate genetic relatedness, a genomic relationship G matrix was constructed using SNP loci (Figure 3B). The results from the G matrix were consistent with the IBS distance matrix, confirming that certain individuals share a notable genetic affinity. The findings underscore the diverse genetic structure of the Beagle dog population, showing that close genetic relationships are limited to a few individuals. These insights are crucial for designing effective breeding management plans and conservation efforts to maintain genetic diversity.

### 3.3. ROH-Based Inbreeding Coefficient Analysis

**Figure 4:**
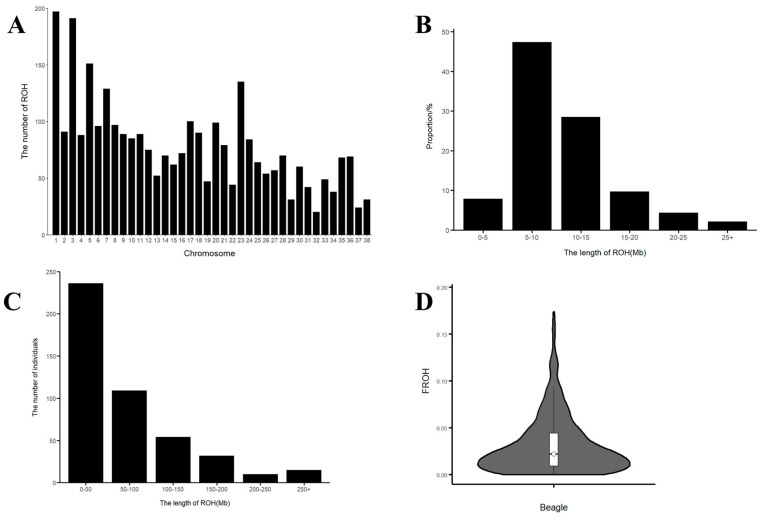
(**A**) The distribution of ROH quantity on each chromosome in Beagle dogs. (**B**) The distribution of ROH length in Beagle dogs. (**C**) The distribution of ROH sample numbers in Beagle dogs. The abscissa represents the length interval of ROH, and the ordinate represents the number of individuals. (**D**) The distribution of the inbreeding coefficient (F_ROH_) based on ROH in Beagle dogs. The violin plot visualizes the data distribution, with the central white dot representing the median. The white box’s edges indicate the upper and lower quartiles.

The detection of ROH number and length in the Beagle dog population was performed using PLINK (v1.90), resulting in the identification of 2990 ROH fragments (Figure 4A). Among these, the fewest ROH fragments (4.99%) were found in the more than 25 Mb category, while the greatest proportion (47.37%) was found in the 5–10 Mb range (Figure 4B). Chromosomes 1 and 3 contained the largest number of ROH fragments, with 197 segments on chromosome 1 and 191 on chromosome 3, while chromosome 32 exhibited the smallest number, with only 20 fragments. The ROH fragment distribution across the remaining chromosomes was less consistent (Figure 4A). This uneven distribution might be linked to a higher occurrence of genetic variation on chromosomes 1 and 3, pointing to the possibility of important genetic traits within these regions. Additionally, the total ROH length per individual was predominantly between 0 and 150 Mb, with an average of 9.55 Mb. A substantial proportion (51.75%) of individuals had a total ROH length between 0 and 50 Mb (Figure 4C). This pattern aligns with the previously observed distribution of ROH lengths, particularly concentrated in the 5–10 Mb range (Figure 4B), which supports the idea of inbreeding in the population. The inbreeding coefficients ranged from 0 to 0.173, with a mean of 0.031 (Figure 4D). In our study, the low average ROH-based inbreeding coefficient (*F_ROH_* = 0.031) and the prevalence of short ROH segments (5–10 Mb) indicate that the observed inbreeding is primarily historical rather than recent. This suggests that past inbreeding events have shaped the genetic structure of the Beagle population, while recent breeding practices have not significantly increased inbreeding levels. Overall, the ROH analysis and inbreeding coefficient assessment indicate a population characterized by relatively low inbreeding and a high degree of genetic diversity.

### 3.4. Cluster Analysis and Family Construction

**Figure 5:**
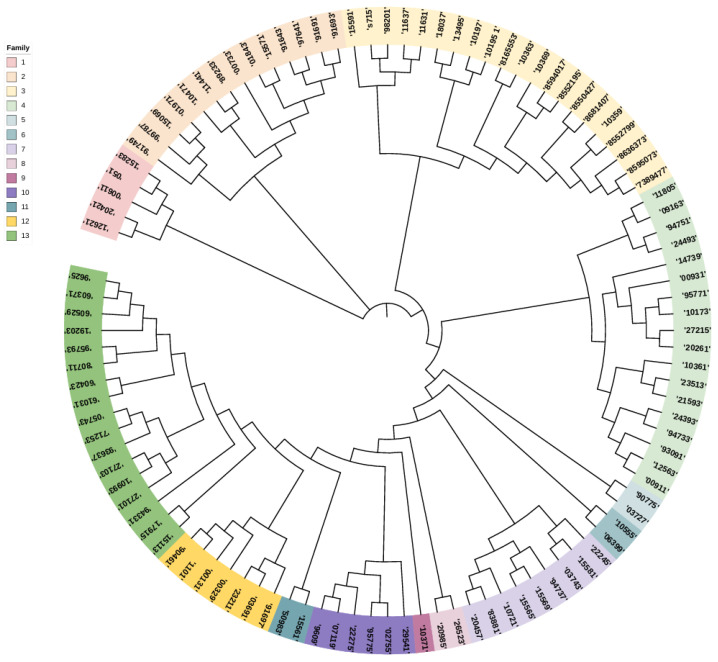
Cluster analysis results for the stud Beagle dogs. The colors in the evolutionary tree are the male samples, and each color represents a family. The numbers in the colors represent the individual ear numbers of the Beagle dogs.

Considering the critical role of stud dogs in genetic conservation, male samples were separately analyzed. A neighbor-joining clustering method was used to evaluate kinship, with a kinship coefficient threshold of ≥0.1. Based on this analysis, 106 stud dogs were classified into 13 distinct lineages, with individuals in the same lineage represented by the same color (Figure 5). The lineages, labeled as Lineage 1 to Lineage 13, contained 5, 14, 21, 18, 2, 2, 9, 2, 1, 6, 2, 7, and 17 individuals, respectively. Among the 13 lineages identified, six lineages contained fewer than 3 individuals, while three lineages had more than 10 individuals, with the largest lineage comprising 21 individuals. This distribution suggests that the breeding strategy may have previously focused on phenotypic traits rather than genetic diversity, leading to an imbalance in the number of individuals across lineages.

### 3.5. Selection Signal Analysis and Functional Annotation

**Figure 6:**
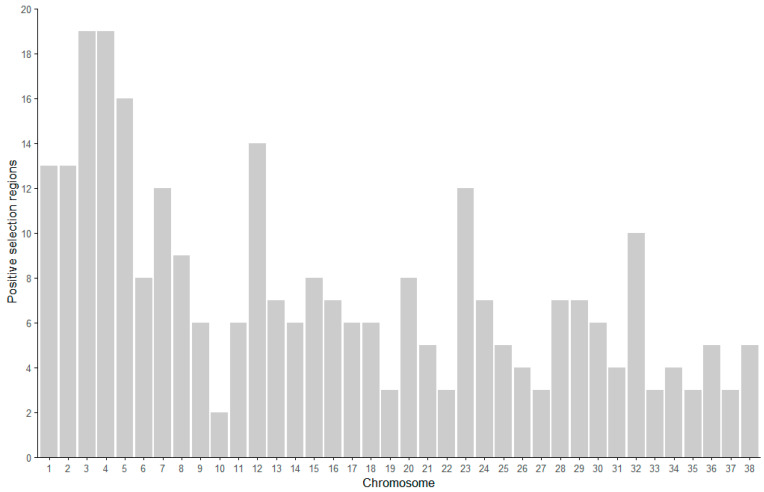
The distribution of positively selected regions on chromosomes in the Beagle population.

Based on the quality control genotype data, the distribution of the genomic regions under positive selection identified through Tajima’s D analysis revealed a total of 284 positively selected regions (Figure 6). These regions were primarily distributed on autosomes, with chromosomes 3 and 4 both containing the highest number of positively selected regions, each having 19. In contrast, chromosome 10 had comparatively fewer regions, with only 2 identified.

The table below lists the top 10 most significant regions under positive selection identified from the Tajima’s D results (Table 3 and Table S1). These regions are primarily distributed across chromosomes 3, 7, 9, 12, 16, 20, 25, 34, and 36. Chromosome 3 contains two regions under positive selection. The region with the smallest Tajima’s D value is located on chromosome 36 and includes four SNPs, while the region on chromosome 20 has the highest number of SNPs, with a total of eight.

**Figure 7:**
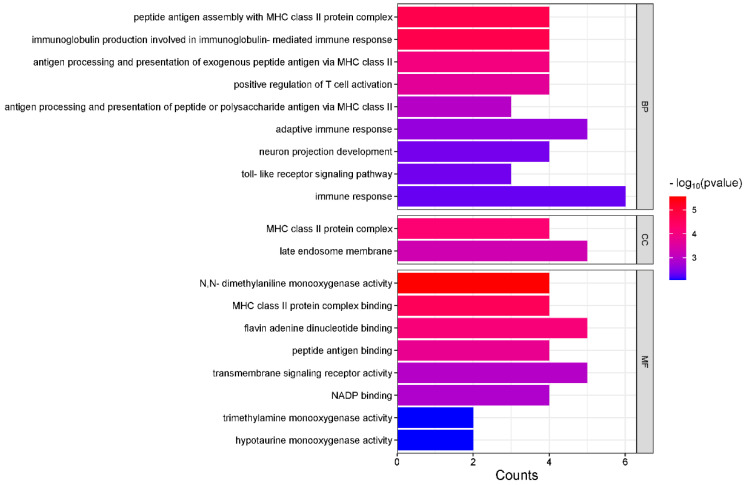
Gene ontology enrichment analysis of candidate genes in Beagle dogs (*p* < 0.01).

Based on the top 10 regions with significant positive selection in the Tajima’s D results, we searched for genes within 500 kb upstream and downstream of each significant region. A total of 94 genes were identified. The genes closest to the significant positive selection regions in terms of physical distance are listed as follows: *TTN*, *CACNA1D*, *SERPINA4*, *TLR5*, *HMCN2*, *ATP13A4*, *DLA-DRA*, *EIF4E2*, *KCNU1*, and *FMO3*. GO functional analysis and KEGG pathway enrichment were conducted on the 94 identified genes using the DAVID database, resulting in 19 GO terms and 10 KEGG pathways (*p* < 0.01). Among the GO terms, 8 were related to molecular functions, 2 were associated with cellular components, and the majority, 9, were involved in biological processes (Figure 7). The most significant GO term (GO:0004499) belongs to the molecular function category and corresponds to N, N-dimethylaniline monooxygenase activity (*p* < 2.7 × 10^−6^). A total of 4 genes are involved in this biological process (Table S2).

**Figure 8:**
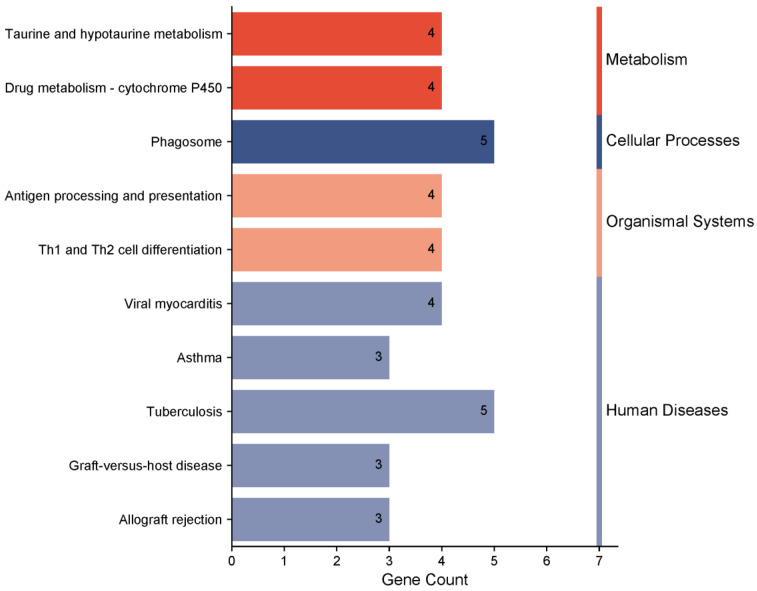
KEGG pathway enrichment analysis of candidate genes in Beagle dogs (*p* < 0.01).

The KEGG pathway enrichment analysis identified 10 significant pathways, with the most prominent being cfa00430: taurine and hypotaurine metabolism. Four genes participate in this pathway (Table S3). Based on the KEGG database, the pathways were categorized into four functional groups: metabolism, cellular processes, organismal systems, and human diseases (Figure 8). These pathways are critical in genetic and biomedical research involving metabolism, cellular physiological processes, systems biology, and disease mechanisms.

## 4. Discussion

Microsatellite markers and single nucleotide polymorphism (SNP) analyses are widely applied in studies of canine genetic diversity [33,34]. Compared to microsatellite markers, SNP chips offer higher accuracy and efficiency, enabling the precise detection of genetic variation among individuals [35]. SNP chips have, thus, become essential tools in breeding programs.

This study utilized the Canine 50K SNP chip to analyze the genetic diversity and population structure of an experimental Beagle breeding population. Key metrics such as MAF, H_O_, H_E_, and PIC are critical indicators of genetic diversity [36]. Research by Choi BH [37] on six native Korean dog breeds revealed that the observed heterozygosity was generally higher than the expected heterozygosity, suggesting a high degree of natural crossbreeding. Similarly, studies by Yang Q on Chinese and Western indigenous dogs [38] found that the expected heterozygosity (0.31) was slightly lower than the observed heterozygosity (0.32). In this study, the foundational Beagle population exhibited an H_O_ of 0.303 and an H_E_ of 0.305. The slightly lower observed heterozygosity (H_O_) relative to the expected heterozygosity (H_E_) suggests that the population may be undergoing genetic optimization, reflected by an increase in genetic purity, although the intensity of artificial selection appears to be relatively low. The proportion of polymorphic markers (P_N_) indicates the percentage of polymorphic loci in the target population [39]. The P_N_ value of 84.9% observed in this study is slightly lower than the 89% reported by Yang Q, reflecting the population’s rich SNP diversity [38]. The average PIC value of 0.305 places the population in the moderate polymorphism category (0.25 < PIC < 0.5) according to Botstein’s criteria [40]. The effective number of alleles (1.513) further supports the population’s high genetic diversity.

Pedigree information is essential in animal breeding, but traditional pedigree-based approaches often fail in large-scale, intensive breeding operations due to errors or missing records [36,41,42]. Genetic distance offers a reliable alternative for accurately determining kinship. In this study, both IBS genetic distance and G matrix analyses indicate that most of the individual Beagles were genetically distant, with only a few exhibiting close relationships. Runs of homozygosity (ROH) serve as key indicators of inbreeding, genetic diversity, and population structure. Additionally, ROH analysis aids in identifying functional genes linked to economically significant traits [43]. The inbreeding coefficient (*F_ROH_*) serves as a key indicator of inbreeding levels. Li Y’s study [15] on Chinese indigenous dogs found significant variation in ROH numbers (482–1809) and lengths (primarily 1–5 Mb), with average *F_ROH_* values ranging from 0.06 to 0.26. In this study, ROH lengths were primarily 5–10 Mb, and the average *F_ROH_* was 0.031, indicating low inbreeding and abundant genetic resources in the Beagle population.

The selection and management of stud dogs are critical for population optimization [44]. In this study, the phylogenetic relationships of 108 male dogs were analyzed, resulting in the construction of a phylogenetic tree that divided them into 13 distinct lineages. Among these, lineages 4, 5, 8, 9, and 11 contained fewer individuals, with Lineage 9 consisting of only one dog. It is recommended to increase the breeding stock of smaller lineages while selecting high-performing individuals from larger lineages for breeding. Continuous adjustments should be made to establish and maintain a rational and stable lineage structure [45].

To further investigate the association between positively selected genomic regions and functional genes within the Beagle population, this study performed selection signal analysis based on Tajima’s D values and incorporated GO and KEGG functional annotations. Several key genes and pathways related to physiological functions and environmental adaptability were identified.

When a specific allele rapidly increases in frequency and becomes fixed in a population, genetic variation in that region decreases because most individuals carry the same allele. Tajima’s D value tends to be negative, as this reduction in diversity leads to lower observed variability. However, it is important to acknowledge that empirical outlier-based approaches, such as Tajima’s D, have inherent limitations. These include sensitivity to demographic history, ascertainment bias from SNP selection, and the inability to distinguish between selection and demographic effects. Integrating complementary methods, such as Fst and XP-EHH, can enhance the robustness of selection signal detection. In the significant Tajima’s D region (−1.200), the *TTN* gene was identified. Research has demonstrated a strong link between this gene and muscle organ development, a critical process for the formation of functional muscle tissue [46]. Komatsu investigated differentially expressed genes in the skeletal muscles of fast- and slow-growing piglets and found that upregulated expression of the *TTN* gene was significantly correlated with increased growth rates [47]. Additionally, Gaar-Humphreys proposed that the TTN gene could be a crucial factor in the development of dilated cardiomyopathy in dogs, emphasizing its significance as a genetic model for human disease [48].

In a significant selection region on chromosome 12, the candidate genes *DLA-DOA*, *DLA-DMA*, and *DLA-DRA* were identified. These genes were notably enriched in immune-related functions, including antigen processing and MHC class II-mediated presentation of exogenous peptide antigens (GO:0019886). This process plays a key role in modulating adaptive immunity, particularly in T-cell activation and antigen presentation. Additionally, key immune-regulatory genes such as *TLR1*, *TLR6*, and *TLR10* were significantly enriched in the toll-like receptor signaling pathway (GO:0002224), indicating their important roles in innate immunity [49,50]. Zhu’s characterization study of the TLR5 gene in dogs revealed that TLR5 is involved in the recognition of bacterial components and participates in the immune response against bacterial pathogens, and it is known that mutations in this gene may affect the immune response of the organism [51].

These immune-related genes were also enriched in multiple disease-associated pathways. For example, *DLA* genes were involved in pathways such as Th1 and Th2 cell differentiation (cfa04658), viral myocarditis (cfa05416), and graft-versus-host disease (cfa05332). These findings indicate that not only are these genes involved in maintaining normal immune system functions, but they may also play critical roles in specific immune-related diseases.

Furthermore, this study revealed significant enrichment of *FMO* family genes (*FMO1*, *FMO2*, and *FMO3*) in multiple metabolic pathways. These genes are involved in taurine and hypotaurine metabolism (cfa00430) and drug metabolism through cytochrome P450 (cfa00982), both of which are essential biological processes. Taurine metabolism, in particular, plays a vital role in maintaining cellular osmotic pressure, antioxidation, and myocardial function, aligning with the Beagle’s utility as a model animal for cardiovascular disease research. Additionally, *FMO1* and *FMO3* are critical for redox reactions, such as trimethylamine oxidation, suggesting their potential roles in regulating energy metabolism and oxidative stress responses.

## 5. Conclusions

This study conducted a comprehensive analysis of genetic diversity and population structure in a foundational Beagle breeding population using the Canine 50K SNP chip. The findings demonstrate substantial genetic diversity within this Beagle population and a low level of inbreeding, with most individuals exhibiting distant kinship. The population displays a diverse family structure, although some lineages have relatively few stud dogs. It is recommended to develop scientifically sound breeding and mating plans to ensure the stability of family structures and the sustainable development of the population. The selection signal analysis pinpointed crucial genes and pathways linked to the physiological functions and environmental adaptability of Beagles, particularly those involved in immune function and disease-related biological processes. This Beagle population has been subject to positive selection for certain traits, particularly those related to immune function, which may enhance their adaptability to various environments. These findings provide valuable support for utilizing Beagles as model animals in cardiovascular disease and immunological studies.

## Figures and Tables

**Table 1:** Results of SNP quality control.

Quality Control Standards	Number of SNP Tags	Number of Beagle Dogs
Total number of SNPs	48,304	459 samples (108 males and 351 females)
SNP with MAF < 0.01	10,925	0
SNP with call rate < 0.90	6181	0
Individuals with call rate < 90%	0	3 samples (2 males and 1 female)
SNPs used after quality control	31,198	456 samples (106 males and 350 females)

**Table 2:** Genetic diversity metrics of Beagle dogs.

Genetic Diversity Parameters	Parameter
MAF	0.224
H_O_	0.303
H_E_	0.305
PIC	0.305
P_N_	0.849
Ae	1.513

**Table 3:** Top 10 regions with significant Tajima’s D selection signals in Beagle dogs.

Chromosome	Start Position (bp)	End Position (bp)	Number of SNPs	Tajima’s D Value	Nearby Gene
36	22,170,000	22,180,000	4	−1.200	*TTN*
20	36,340,000	36,350,000	8	−1.031	*CACNA1D*
9	53,550,000	53,560,000	2	−0.934	*SERPINA4*
3	73,540,000	73,550,000	2	−0.881	*TLR5*
3	84,580,000	84,590000	3	−0.873	*HMCN2*
34	25,100,000	25,110,000	3	−0.868	*ATP13A4*
12	2,160,000	2,170,000	2	−0.863	*DLA-DRA*
25	44,160,000	44,170,000	2	−0.863	*EIF4E2*
16	28,450,000	28,460,000	2	−0.846	*KCNU1*
7	27,560,000	27,570,000	2	−0.845	*FMO3*

## Data Availability

The original contributions presented in this study are included in the article/Supplementary Material. Further inquiries can be directed to the corresponding authors.

## Supplementary Materials

The following supporting information can be downloaded at: https://www.mdpi.com/article/10.3390/genes16040358/s1, Figure S1: The MAF distribution plot; Table S1. Candidate genes within the top 1% of positive selection regions; Table S2: Gene Ontology Enrichment Analysis of Candidate Genes in Beagle Dogs (*p* < 0.01); Table S3: KEGG Pathway Enrichment Analysis of Candidate Genes in Beagle Dogs (*p* < 0.01).
