# Supplementary figures and images for: An Analysis of the Genetic Diversity, Genetic Structure, and Selection Signal of Beagle Dogs Using SNP Chips
Source: Genes (Basel). 2025 Mar 21;16(4):358. doi: 10.3390/genes16040358 (PMC12026597; doi:10.3390/genes16040358)

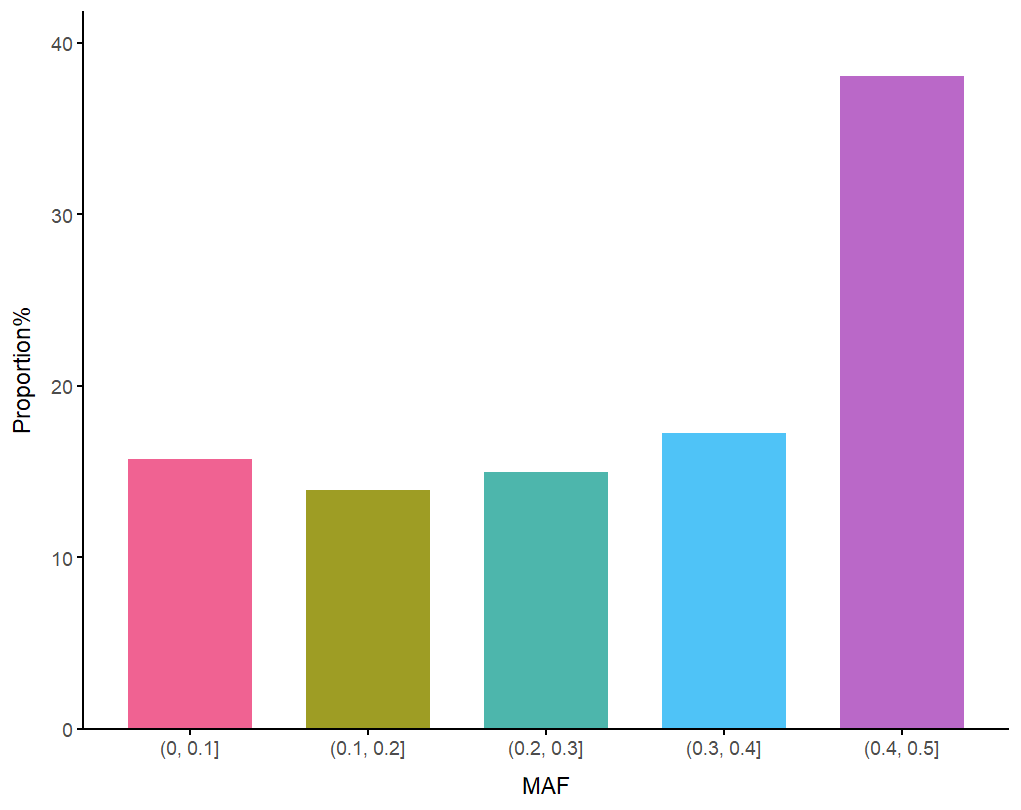

Supplement: Supplementary file 1 [file genes-16-00358-s001.zip › Figure S1.png]
